# 

**Published:** 1883-12

**Authors:** 


					﻿SSTABXuTFiTT-F;r> IIST 18B5.
Ox» hkrikh.	TbVrPWniTD 1RRR	Nkw Bekos
Vol. XXiyNo. ».	UXjVXjIHAJXjAX,, AOOO.	Vol. Ill—No. 8
ATLANTA
MEDICAL REGISTER.
(ATLANTA MEDICAL AND SURGICAL JOURNAL.)
EDITED SV
J. H. LOGAN, A.M., M.D.
R. H> piCKSON, PUBLISHER. TERMS, $1.50 A YEAR IN ADVANCE
ATLANTA, GEORGIA:
B. B. DICKSON, BOOK AND JOB PRINTER AND BOOKBINDER.
1883.
				

